# The association between dietary vitamin C intake and periodontitis: result from the NHANES (2009–2014)

**DOI:** 10.1186/s12903-022-02416-7

**Published:** 2022-09-08

**Authors:** Wei Li, Jukun Song, Zhu Chen

**Affiliations:** 1 Medical College of Guizhou University, Guiyang, Guizhou, 550025 China; 2 Department of Oral and Maxillofacial Surgery, School of Stomatology, Guizhou Medical University, Guiyang, Guizhou, 550001 China; 3 Zunyi Medical University, Guiyang Hospital of Stomatology, Guiyang, Guizhou, 550005 China

**Keywords:** Periodontitis, Dietary vitamin C intake, NHANES database

## Abstract

**Background:**

The purpose of this study was to investigate whether periodontitis is associated with dietary vitamin C intake, using data from The National Health and Nutrition Examination Survey (NHANES) 2009–2014.

**Methods:**

The study included 5145 adults (age ≥ 30 years) with periodontitis as a dichotomous variable and daily intake of vitamin C as a continuous variable. Multiple sets of covariates, such as age, sex, number of flossing, etc., were selected. Using EmpowerStats version 3.0, multivariate logistic regression analysis and hierarchical analysis were performed on the data, and curve fitting graphs were made.

**Results:**

There were no statistically significant differences (*P* > 0.05) between the four dietary vitamin C intake groups (quartiles, Q1–Q4) and covariates (drinking alcohol and hypertension). The low VC intake group (Q1) was more prone to periodontitis than Q2, Q3, and Q4 (all OR < 1.00). A threshold nonlinear association was found between vitamin C (mg) log10 transformation and periodontitis in a generalized additive model (GAM) (*P* = 0.01).

**Conclusion:**

The relationship between dietary vitamin C intake and the likelihood of periodontitis was non-linear. The smallest periodontitis index occurred when dietary vitamin C intake was 158.49 mg. Too little or too much vitamin C intake increases periodontitis.

## Introduction

More than half of the world's population suffers from various degrees of periodontal disease (including periodontitis and gingivitis), and more than 10% suffer from severe periodontitis [1], which is the first cause of tooth loss in adults over 35 years old. Periodontitis is an infectious periodontal disease caused by a combination of factors, and its clinical manifestations also have some comorbidities, such as liver diseases [2], diabetes [3], atherosclerosis [4], and so on. Therefore, in addition to mechanical removal of plaque and other related operations during the treatment process, drugs and diet should also be taken to continuously treat the patient's later oral problems. Vitamin C (VC) in the diet occupies a very important place, and many studies have proved that the physiological role of vitamin C can prevent or treat people suffering from periodontitis [5].

Vitamin C is a polyhydroxy compound with a structure similar to glucose [6]. The two adjacent enol hydroxyl groups on the 2nd and 3rd positions in the molecule are easily dissociated to release H + , so it has the property of acid, also known as L-ascorbic acid. Vitamin C has many physiological effects, including depigmentation of melanin pigmentation spots on the skin and gums [7], improved oxidative stress and immune regulation [8], and even improved bone health and tendons [9]. A small number of scholars have studied the relationship between vitamin C and periodontitis and found that low intake people are more prone to periodontitis. This study differs from, first, the increased sample size of the study population and more confounding factors for periodontal or systemic health. Second, the daily intake of vitamin C in the diet and the likelihood of periodontitis were plotted as a curve-fitted graph for analysis. We conducted a cross-sectional study using data from NHANES dataset (2009–2014). The aim was to examine the relationship between daily dietary vitamin C intake and periodontitis, and to assess whether this relationship was influenced by potential confounding factors, and whether meaningful curve inflection points could be found in their curve-fit plots, which this part of the clinical research will provide theoretical basis.

## Materials and methods

### Data source

Data from the United States NHANES collected from 2009 to 2014 were used for this analysis (http://wwwn.cdc.gov/nchs/nhanes). The National Health and Nutrition Examination Survey (NHANES) is a program of studies designed to assess the health and nutritional status of adults and children in the United States. The NHANES interview includes demographic, socioeconomic, dietary, and health-related questions. The examination component consists of medical, dental, and physiological measurements, as well as laboratory tests administered by highly trained medical personnel. Inclusion criteria is as follow: 1. NHANES participants 30 years of age and older. 2. NHANES participants with VC data in the Daily Total Energy and Nutrient Intake from Food and Beverage Survey. 3. NHANES participants with periodontal examinations in the oral health examination section. Exclusion criteria was listed as follow: 1. Participants who did not undergo a complete periodontal examination and who received ineffective vitamin C on the first day of dietary data. 2. A very small number of participants with missing covariates such as education, marriage, hypertension, and sleep disorders.

### Definition of periodontal disease

Periodontitis data were obtained from Oral Health-Periodontal in Examination Data of NHANES 2009–2014. This periodontal oral health data will be used to assess the prevalence of major oral health diseases and conditions, including dental caries, periodontal disease, and dental fluorosis. Survey participants aged 30 and older who had at least one tooth (excluding third molars) and did not meet any of the health exclusion criteria were eligible for periodontal evaluation.

In this study, we used the criteria proposed by Eke et al. [10]. The CDC-AAP definition is based on attachment loss (AL) and pocket depth (PD) measurements at four interproximal sites per tooth, and periodontitis is therefore divided into three categories: mild, moderate, and severe. Mild periodontitis was defined as 2 interproximal sites with AL ≥ 3 mm, and ≥ 2 interproximal sites with PD ≥ 4 mm (not on the same tooth) or one site with PD ≥ 5 mm. Moderate periodontitis was defined as 2 interproximal sites with AL ≥ 4 mm (not on the same tooth), or ≥ 2 interproximal sites with PD ≥ 5 mm (not on the same tooth). Severe periodontitis was defined as 2 interproximal sites with AL ≥ 6 mm (not on the same tooth) and ≥ 1 interproximal site with PD ≥ 5 mm. Therefore no periodontitis was no evidence of mild, moderate, and severe periodontitis.

### Dietary vitamin C intake

For each NHANES participant, total daily energy and nutrient intake from food and beverages, and whether food intake was normal, much more or less than usual, were included in the total Nutrient intake file. For this study, we chose VC (mg) on the first day's total nutrient intake. It is a 24-h first meal recall interview collected in person by the Mobile Examination Center (MEC).

### Study confounders

We comprehensively screened 16 confounding factors that may be related to periodontitis and VC, namely age, gender, race, education, marital status, BMI, annual family income, Diabetes, hypertension, hypercholesterolemia, sleep disturbance, smoking, alcohol consumption, oral health, frequency of flossing, and energy have all been identified as risk factors, as described in the relationship between smoking cessation time and periodontitis in former smokers, alcohol intake and periodontitis, oral health and diabetes, periodontitis and sleep duration [11–16].

The age range was from 30 to 80 years old. Ethnicity was Mexican American and Other Hispanic, Non-Hispanic White, Non-Hispanic Black, and Other Race—including Multi-Racial. Education was considered less than high school, high school, and more than high school. Marital status was divided into married, widowed/divorced, and single [17]. Annual household income is categorized as over $20,000 and under $20,000. Participants in the rest of the variables were distinguished by yes or no.

### Statistical analysis

**Fig. 1:**
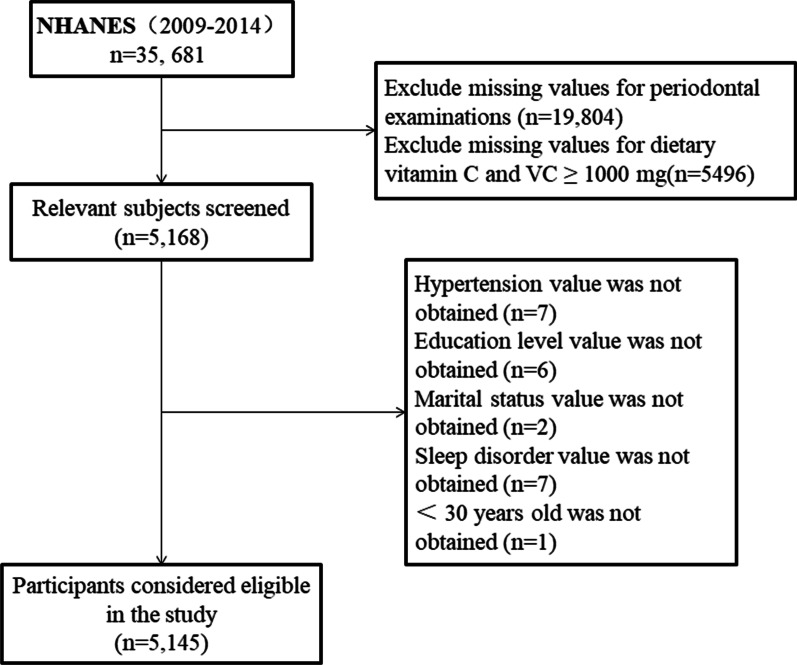
Flow chart of procedures from identification of eligible patients to final inclusion

We utilize EmpowerStats version 3.0 (http://www.empowerstats.net/analysis) to merge data from the NHANES (2009–2014) database. The flowchart for detailed screening of eligible participants is shown in Fig. 1. Periodontitis was classified as a dichotomous variable, with or without periodontitis. VC as a continuous variable is represented by quartiles. Continuous variables among covariates are presented as mean ± standard deviation, and categorical variables are presented as percentages.

This study aimed to investigate whether dietary VC intake was associated with periodontitis in selected participants. Using EmpowerStats version 3.0, we present the distribution of baseline data for patients enrolled in this study across different dietary protein intake groups (quartiles). The chi-square test or Student's t-test was used to test the distribution's *P*-value. Second, we employed weighted univariate and multivariate linear regression models. Four statistical models were established: Model I, with no adjustment for covariates; Model II, with adjustment for conventional variables of age, sex, and race selected; and Model III, with all covariates shown in Table 1. Finally, a smooth curve fitting graph was made between VC and the possibility of suffering from periodontitis. The result of the exploration is a nonlinear relationship, and we analyzed the threshold effect and saturation effect on it. The model I in the threshold analysis is a linear relationship, and the effect size is obtained; model II is a non-linear relationship, and the effect size of the inflection point and the segment is obtained. The log-likelihood ratio test was used to verify the difference between model I and model II.

**Table 1:** Baseline Characteristics of participants with different Vitamin C Intakes

Characteristics	Vitamin C(mg)				*P* value
	Q1 (0.00–21.70)	Q2(21.80–54.50)	Q3(54.60–113.00)	Q4 (113.10–964.70)	
N	1284	1283	1290	1288	
Age(mean ± SD, years)	50.02 ± 13.96	52.30 ± 13.86	53.49 ± 14.47	52.24 ± 14.13	< 0.001
BMI (mean ± SD, Kg/m**2)	29.96 ± 7.29	29.43 ± 6.33	28.99 ± 6.18	29.02 ± 6.58	< 0.001
Energy (kcal)	1881.67 ± 904.96	2052.55 ± 899.27	2100.29 ± 939.44	2359.72 ± 1053.54	< 0.001
*Had at least 12 alcohol drinks/one year? (%)*					0.924
Yes	906 (70.56%)	906 (70.62%)	884 (68.53%)	902 (70.03%)	
No	310 (24.14%)	313 (24.40%)	335 (25.97%)	316 (24.53%)	
Not recorded	68 (5.30%)	64 (4.99%)	71 (5.50%)	70 (5.43%)	
*Hypertension(%)*					0.176
Yes	478 (37.23%)	499 (38.89%)	513 (39.77%)	462 (35.87%)	
No	806 (62.77%)	784 (61.11%)	777 (60.23%)	826 (64.13%)	
*Hyperlipidemia(%)*					0.006
Yes	428 (33.33%)	516 (40.22%)	475 (36.82%)	475 (36.88%)	
No	676 (52.65%)	633 (49.34%)	671 (52.02%)	669 (51.94%)	
Not recorded	180 (14.02%)	134 (10.44%)	144 (11.16%)	144 (11.18%)	
*Sex(%)*					0.006
Male	631 (49.14%)	627 (48.87%)	607 (47.05%)	692 (53.73%)	
Female	653 (50.86%)	656 (51.13%)	683 (52.95%)	596 (46.27%)	
*Race(%)*					< 0.001
Mexican American and Other Hispanic	306 (23.83%)	318 (24.79%)	327 (25.35%)	357 (27.72%)	
Non-Hispanic White	593 (46.18%)	623 (48.56%)	592 (45.89%)	527 (40.92%)	
Non-Hispanic Black	287 (22.35%)	221 (17.23%)	235 (18.22%)	253 (19.64%)	
Other Race—including Multi-Racial	98 (7.63%)	121 (9.43%)	136 (10.54%)	151 (11.72%)	
*Education level(%)*					< 0.001
Less than high school	354 (27.57%)	299 (23.30%)	293 (22.71%)	279 (21.66%)	
High school	342 (26.64%)	279 (21.75%)	260 (20.16%)	206 (15.99%)	
More than high school	588 (45.79%)	705 (54.95%)	737 (57.13%)	803 (62.34%)	
*Marital status(%)*					0.446
Married	819 (63.79%)	847 (66.02%)	851 (65.97%)	859 (66.69%)	
Widowed/divorced	320 (24.92%)	301 (23.46%)	314 (24.34%)	283 (21.97%)	
Single	145 (11.29%)	135 (10.52%)	125 (9.69%)	146 (11.34%)	
*Annual household income(%)*					< 0.001
< $20,000	286 (22.27%)	230 (17.93%)	222 (17.21%)	176 (13.66%)	
≥ $20,000	955 (74.38%)	991 (77.24%)	1013 (78.53%)	1049 (81.44%)	
Not recorded	43 (3.35%)	62 (4.83%)	55 (4.26%)	63 (4.89%)	
*Diabetes history(%)*					< 0.001
Yes	174 (13.55%)	176 (13.72%)	179 (13.88%)	121 (9.39%)	
No	1076 (83.80%)	1080 (84.18%)	1067 (82.71%)	1140 (88.51%)	
Not recorded	34 (2.65%)	27 (2.10%)	44 (3.41%)	27 (2.10%)	
*General health condition(%)*					0.010
Healthy	939 (73.13%)	987 (76.93%)	1000 (77.52%)	1009 (78.34%)	
Unhealthy	345 (26.87%)	296 (23.07%)	290 (22.48%)	279 (21.66%)	
*Number of flossing/a week(%)*					< 0.001
0	486 (37.85%)	407 (31.72%)	394 (30.54%)	351 (27.25%)	
1	80 (6.23%)	98 (7.64%)	96 (7.44%)	83 (6.44%)	
2	133 (10.36%)	108 (8.42%)	125 (9.69%)	120 (9.32%)	
3	105 (8.18%)	116 (9.04%)	101 (7.83%)	124 (9.63%)	
4	65 (5.06%)	78 (6.08%)	63 (4.88%)	70 (5.43%)	
5	35 (2.73%)	49 (3.82%)	62 (4.81%)	49 (3.80%)	
6	20 (1.56%)	10 (0.78%)	13 (1.01%)	17 (1.32%)	
7	337 (26.25%)	408 (31.80%)	428 (33.18%)	461 (35.79%)	
Not recorded	23 (1.79%)	9 (0.70%)	8 (0.62%)	13 (1.01%)	
*Sleep disorder(%)*					0.006
Yes	127 (9.89%)	141 (10.99%)	104 (8.06%)	96 (7.45%)	
No	1157 (90.11%)	1142 (89.01%)	1186 (91.94%)	1192 (92.55%)	
*Smoked at least 100 cigarettes in life(%)*					0.924
Yes	906 (70.56%)	906 (70.62%)	884 (68.53%)	902 (70.03%)	
No	310 (24.14%)	313 (24.40%)	335 (25.97%)	316 (24.53%)	
Not recorded	68 (5.30%)	64 (4.99%)	71 (5.50%)	70 (5.43%)	

## Results

### Baseline characteristics of participants

Baseline characteristics of participants in this study by quartile of dietary VC intake are shown in Table 1. The distributions of alcohol consumption, hypertension, marital status and smoking were not significantly different among the four VC intake groups (quartiles, Q1–Q4) (*p* value > 0.05). In the Q1 group, participants with lower VC intake were predominantly married and non-Hispanic whites. Compared with the other three groups, this group was characterized by a lower education level, having a lower income, using less dental floss, being widowed or divorced, lower energy intake and poorer health.

### Dietary vitamin C intake and periodontitis levels

The correlation between dietary VC intake and periodontitis is presented in Table 2. The OR values of dietary VC intake as a continuous variable and periodontitis were all 1 and were not significant. Dietary VC intake was then stratified into categorical variables by quartile, and *P* for trend was estimated. In the unadjusted model, the low VC intake group (Q1) was more likely to have the risk of periodontitis than Q2, Q3, and Q4 (all OR < 1.00), and the trend *P*-value was 0.0367. In Adjust I model, three conventional variables of age, gender, and race were adjusted. In Adjust II model, all covariates listed in Table 1 were adjusted. The OR value results of these two models were consistent with the trend of the OR value of the unadjusted model group, which indicated that too low VC intake would be more prone to periodontitis. What is more noteworthy is that the OR value of the Q3 group (OR = 0.78, *P* = 0.0062) in Adjust II model is smaller than that of the Q1 group (1.00), but the P for trend of Model I was < 0.0001, while that of Model II was 0.1345. This trend in the respective OR effect sizes of the quartiles revealed a possible non-linear relationship between dietary VC intake and periodontitis.

**Table 2:** Relationship between Vitamin C and periodontitis in different models

Exposure	Non-adjusted	Adjust I	Adjust II
Vitamin C(mg)	1.0 (1.0, 1.0) 0.774	1.0 (1.0, 1.0) 0.162	1.0 (1.0, 1.0) 0.648
*Vitamin C(quartile)*			
Q1	1.0	1.0	1.0
Q2	0.93 (0.79, 1.08) 0.3335	0.85 (0.72, 1.00) 0.0569	0.98 (0.82, 1.17) 0.8165
Q3	0.80 (0.68, 0.93) 0.0046	0.68 (0.57, 0.80) < 0.0001	0.78 (0.65, 0.93) 0.0062
Q4	0.88 (0.76, 1.03) 0.1142	0.73 (0.62, 0.87) 0.0003	0.93 (0.78, 1.12) 0.4593
*P* for trend	0.0367	< 0.0001	0.1345

Non-adjusted model adjust for: None

Adjust I model adjust for: Age; Sex; Race

Adjust II model adjust for: Age; Sex; Race; Had at least 12 alcohol drinks/one year?; BMI; Hypertension; Hyperlipidemia; Education level; Marital status; Annual household income; Diabetes history; General health condition; Number of flossing/a weeks; Sleep disorder; Smoked at least 100 cigarettes in life; Energy

### Exploration of nonlinear relationships

**Fig. 2:**
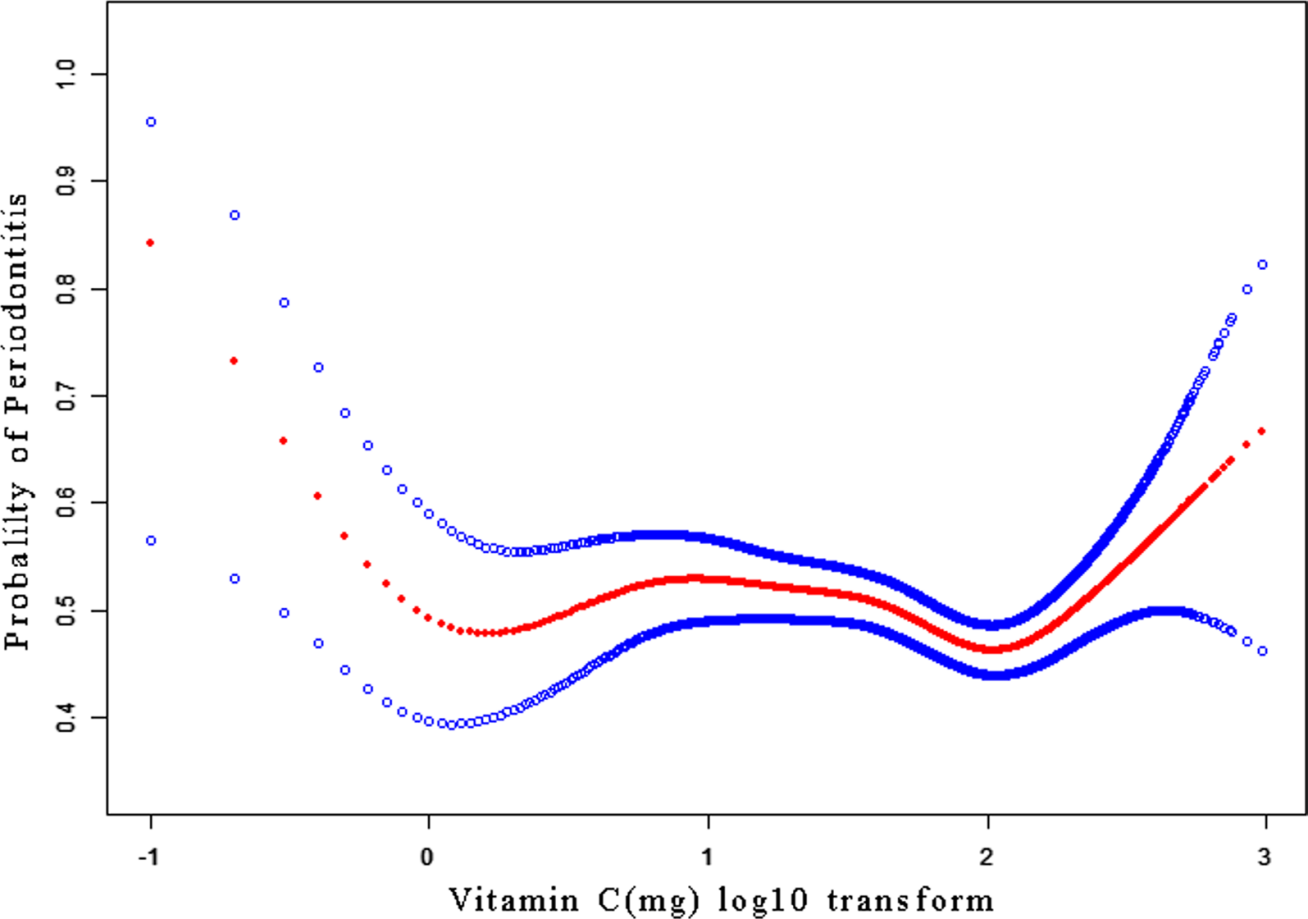
Association between Vitamin C and Periodontitis.Noted: A threshold, nonlinear association between Vitamin C (mg) log10 transform and Periodontitis was found (*P* = 0.01) in a generalized additive model(GAM). A solid rad line represents the smooth curve fit between variables. Blue bands represent the 95% of confidence interval from the fit. All adjusted for Age; Sex; Race; Had at least 12 alcohol drinks/one year?; BMI; Hypertension; Hyperlipidemia; Education level; Marital status; Annual household income; Diabetes history; General health condition; Number of flossing/a weeks; Sleep disorder; Smoked at least 100 cigarettes in life; Energy

According to the results of regression analysis, we used log10 transformed data for further processing due to the skewed distribution of dietary VC intake. A curve fitting analysis was performed between the log10 transformation of dietary VC intake and the likelihood of periodontitis, and the results are shown in Fig. 2.

At the same time, the linear regression model and the two-segment linear regression model were compared, and the log-likelihood ratio test *P* = 0.003, the results are shown in Table 3. This indicates that a two-piece linear regression model should be used to fit the model. The inflection point was calculated to be 2.2 (log10 transformation) by a two-segment linear regression model and recursive algorithm. Confidence intervals for thresholds were determined by the Bootstrap resampling method. There was a certain U-shaped curve between dietary VC intake and periodontitis, and there was a threshold effect, and the threshold for VC intake was 158.49 mg (the inflection point was 2.2). To the left of the inflection point, the effect size and 95% confidence interval was 0.8 (0.7, 1.0), which means that each unit increase in dietary VC intake was associated with a 20% reduction in the risk of periodontitis index, which was significant (*P* = 0.0094). Similarly, on the right side of the inflection point, the effect size and 95% CI and P value were 3.1 (1.4, 7.0), respectively, revealing that each unit increase in dietary VC intake was associated with a 2.7-fold increase in the risk of periodontitis index (*P* = 0.0059).

**Table 3:** The results of the two-piecewise linear regression model

Outcome	Periodontitis	
	OR (95%CI)	*P* value
Fitting by weighted linear regression model	0.9 (0.8, 1.0)	0.1355
*Fitting by the weighted two-piecewise linear regression model*		
Inflection point	2.2	
< 2	0.8 (0.7, 1.0)	0.0094
> 2	3.1 (1.4, 7.0)	0.0059
Log-likelihood ratio test	0.003	

Independent variable is Dietary Vitamin C intake (mg) log10 transform; the dependent variable is Periodontitis (binary variable); Covariates involved in this model were the same as presented in Fig. 2

## Discussion

Oral health has always been a global public health issue, closely related to people's living standards and social factors. Periodontitis became the sixth most prevalent disease in humans as early as 2010 [18]. Therefore, studies on the etiology of periodontitis, related inflammatory factors and biomarkers released during periodontitis have become the theoretical basis for clinical treatment. For example, Matarese G [19] demonstrated that transglutaminase 2 (TG2) may be involved in the molecular mechanism of inflammatory response in periodontal disease. They experimentally evaluated the lower expression of TG2 in cultured normal human than in patient-derived human periodontal ligament (HPDL) cells, its nuclear factor-kappaB (NF-κB) receptor activator ligand (RANKL)/secretory bone The ratio of protectin (RANKL/OPG) was also relatively low, and the ratio of RANKL/OPG in HPDL cells of patients with periodontitis was positively correlated with the expression level of TG2 gene. Ferlazzo N [20]et al. conducted methylation studies by extracting genomic DNA from saliva samples from patients with oral squamous cell carcinoma (OSCC) and healthy controls, and found that p16 and O6-methylguanine-DNA methyltransferase (MGMT) gene promoters in the patient group The methylation frequencies of the methylenetetrahydrofolate reductase (MTHFR) gene polymorphisms were significantly associated with their methylation. It indicated that hypermethylation of OSCC-related genes may be affected by MTHFR gene polymorphisms. In addition, Isola G [21]et al. investigated the correlation between serum and saliva concentrations of NOD-like receptor family pyrrole domain-containing protein-3 (NLRP3) in patients with periodontitis and type 2 diabetes mellitus (DM). The results showed that the serum and saliva concentrations of NLRP3 in the periodontitis patient group and the periodontitis + type 2 DM group were significantly higher than those in the healthy control group and the type II DM group, and NLRP3 was proven to be an important predictor of periodontitis disease. People detect early biomarkers of inflammation such as MTHFR, NLRP3, TG2, which can reduce the risk of periodontal inflammation. In many studies related to periodontitis, in addition to the expression of these genes and proteins and the level of inflammatory factors, some energy, vitamins, etc. that people consume in their diets have also attracted the attention of researchers.

In articles related to periodontitis, the possible effect of dietary VC intake has long been noted and researched [5, 22]. Studies have pointed out that people with low VC intake are more prone to periodontitis. In the study by Lee, Luo et al., [23, 24] which also treated dietary VC intake by quartiles or quintiles, the result was that insufficient intake of VC was significantly associated with periodontitis.

In a study on VC and periodontitis, Johan P Woelber [25] et al. set up an experimental group rich in vitamin C, D, etc. on a daily diet, and the experimental group showed significantly less gingival bleeding than the control group. A related case study [26] pointed out that VC can be used as an anti-inflammatory agent to treat chronic gingival inflammation because they injected VC into gingival tissue for clinical observation and found that the inflammation at the injection site was significantly improved. In experiments in mice, Ayşe Toraman et al. [27] found that topical treatment of VC was beneficial in reducing serum C-terminal terminal peptide fragment (CTX) and gingival matrix metalloproteinase (MMP)-8 levels while acting as an immunomodulatory agent to reduce diabetes on teeth Adverse effects of weekly tissue. Gaetano Isolaet al. [28] studied the effect of periodontitis on saliva and serum VC levels, and the results showed that saliva and serum VC were significantly lower in periodontitis patients compared with controls. These indicate that VC and periodontitis have mutual effects. For mechanistic studies, studies [29] have investigated the effect of ascorbic acid (AA) on inflammation in lipopolysaccharide (LPS-G) treated oral stem cells. The results indicated that AA regulates the inflammatory process through the NFκB/Caspase-1/IL-1*β* pathway.

The advantage of this study over other similar studies is that it discussed not only the baseline characteristics of participants with different VC intakes but also a smooth curve fit between VC intake and periodontitis. Second, the sample size was large, including 9937 participants, providing strong evidence for a quantitative assessment of the association between dietary VC intake and periodontitis index levels. The optimal value of VC intake obtained in this study was 100 mg, which was also calculated by some scholars using kinetic models in the early years [30]. However, in a newer oral-related article, the optimal plasma VC level was 56.8 μmol/l, and people needed to consume at least 200 mg of VC per day to achieve it [31]. It is recommended that everyone obtain VC through daily consumption of fruits and vegetables. Some scholars also recommend the dietary allowance (RDA) of vitamin C to be 75 mg for women and 90 mg for men, because a high intake of VC is sometimes accompanied by gastrointestinal discomfort [32].

But for the existing results, we should also consider the following three points. First, the study was a cross-sectional study with certain limitations. The results of this study can only provide etiological clues for further research on the relationship between dietary VC intake in daily meals and periodontitis, and the study on supplemental VC intake was not involved. Secondly, the NHANES of the data source is the statistics of the National Center for Health Statistics (NCHS), so it will be subject to geographical restrictions. Third, dietary VC intake in this study was a 24-h dietary review interview with participants.

## Conclusions

Our data show that the relationship between dietary VC intake and the likelihood of periodontitis is non-linear. The periodontitis risk index was the smallest when the dietary VC intake was 158.49 mg. Too little or too much VC intake will increase the risk of periodontitis, for the reason that people over the age of 30 should adequate intake of dietary VC and pay attention to nutritional balance in their daily diet.

## Data Availability

The datasets generated and/or analyzed for this current study are not publicly available to protect participant confidentiality and are considered restricted meta data for administrative purposes and quality control. Data can be downloaded from the ‘ NHANES’ database (https://www.cd c.gov/nchs/nhan es/index.htm).

## Abbreviations

NHANES: The national health and nutrition examination survey
VC: Vitamin C
AL: Attachment loss
PD: Pocket depth
MEC: Mobile examination center
GAM: Generalized additive model
CTX: C-terminal terminal peptide fragment
MMP: Matrix metalloproteinase
AA: Ascorbic acid
LPS-G: Lipopolysaccharide
RDA: Recommend the dietary allowance
NCHS: The national center for health statistics
